# Base-catalysed decomposition of polychlorinated biphenyls in transformer oils by mixture of sodium hydroxide, glycerol and iron

**DOI:** 10.1098/rsos.172401

**Published:** 2018-06-13

**Authors:** Milad Akhondi, Ali A. Dadkhah

**Affiliations:** Department of Chemical Engineering, Isfahan University of Technology, Isfahan 84156-83111, Iran

**Keywords:** base-catalysed decomposition, PCBs, sodium hydroxide, glycerol, transformer oil

## Abstract

In this research, a method based on base-catalysed decomposition (BCD) was developed using sodium hydroxide and glycerol for dechlorination of transformer oils with low levels of polychlorinated biphenyls (PCBs). PCB removal and dechlorination efficiencies were measured by gas chromatography and the silver nitrate titration method, respectively. PCB dechlorination and removal efficiencies after 4 h at 250°C and in the presence of iron were 97.4% and 99.8%, respectively. However, in the absence of iron the same efficiencies were calculated to be 79.4 and 99.7%, respectively. The dielectric loss factor of oil refined at 250°C was 0.0064 and did not meet the required standards to be reused in the transformers. However, that refined at 200°C satisfied the standards with acceptable conversion rates. Use of iron, besides increasing conversion rates, changed the mechanism of the reaction from nucleophilic substitution to a combination of hydrodechlorination and nucleophilic substitution. In the presence of iron, highly chlorinated PCBs were converted to less chlorinated PCBs, and this caused the concentration of less chlorinated PCBs to reach a peak and then decline thereafter. The production of PCB 36 confirmed this. However, in the absence of iron particles, all changes in the PCB concentration curves were downward.

## 1. Introduction

More than four decades have passed since the production and use of polychlorinated biphenyls (PCBs) were banned, but they remain a big problem for human and environmental health [1]. PCBs are chlorine-bearing derivatives of biphenyl with 1–10 chlorine atoms and have many industrial applications due to their dielectric characteristics, chemical stability at high temperatures, insulating properties and low vapour pressure [2]. PCBs were mainly used in the production of Askarel, which was produced in the reaction between Arochlor, a trade name for a mixture of PCBs in the USA, and trichlorobenzene. They were used as the dielectric fluid in capacitors, transformer oils, hydraulic fluids, etc. [2,3]. According to the Stockholm Convention on Persistent Organic Pollutants (POPs), PCBs were placed in the group of POP Materials due to their destructive effects on the human body, their persistence in the environment and their bioaccumulation in the food chain. Member States of this convention are bound to destroy gradually their PCB stockpiles by 2025 [4]. Incineration is the oldest and most reliable method for PCB disposal [5]. For destruction of PCBs, although in recent years new methods have been developed, such as using an electron beam accelerator [6], plasma [7], lasers [8], microwaves [9,10], adsorption [11] and absorption [12], yet most studies have focused on chemical methods [13]. One of the practical and relatively inexpensive methods of purifying transformer oils and treating soil polluted with PCBs that is mostly used in developed countries is base-catalysed decomposition (BCD) [14,15]. In this method, PCBs are dechlorinated by using a high-boiling-point hydrocarbon, base and metal catalyst. Bases as nucleophilic activation groups, hydrocarbons as hydrogen donors and a metal catalyst as an electron transfer surface take part in the PCB dechlorination process [16,17]. Various mechanisms have been proposed for BCD. Ryoo *et al*. [18] found that reaction between polyethylene glycol (PEG) and PCBs is carried out by nucleophilic substitution in the presence of alkaline hydroxide. Liu *et al*. [19] considered hydrogen free radicals produced from decomposition of PCBs during thermal treatment as a factor for dechlorination. Xiao *et al*. [20] proposed a combination of mechanisms in which nucleophilic substitution was the major mechanism and hydrodechlorination was the minor mechanism of PCB decomposition.

In previous studies, performance of various chemical methods using sodium bicarbonate [21], fly ash, alcohols and metal catalyst [22], quicklime and iron [23], sodium hydroxide, paraffin oil and iron [16], PEG and sodium hydroxide [20], nucleophile materials and sodium hydroxide [24] and sodium carbonate and glycerol [17] has been investigated. Methods in which paraffinic or aliphatic oils are used require high temperatures. However, techniques that use alcohols are more efficient and are low-temperature processes, but, besides these positive aspects, most alcohols are toxic or expensive [4,13,17]. Glycerol is a trihydric alcohol which is compatible with most chemicals, does not have harmful effects on the environment, has a high melting point, and can play the roles of hydrogen donor and solvent in the BCD process [17,25]. Glycerol is expensive, but raw glycerol obtained from sources such as the biodiesel production process can be used in the BCD process [17,26]. Most research concerning PCB dechlorination have been conducted on pure PCBs, or on heavily polluted transformer oils, and few studies have been reported on transformer oils contaminated with low levels of PCBs (50–500 ppm). When new PCB-free oil circulates inside an old transformer which was previously operating with Askarel, then the oil becomes polluted with a low concentration of PCBs. Physical, chemical and electrical characteristics of transformer oils after dechlorination is another issue not dealt with in research carried out in this area.

In this research, for the first time glycerol is used as a solvent and hydrogen source for dechlorination of PCBs in lightly polluted transformer oils, and the effect of several factors such as temperature, time and reactants are investigated. Moreover, the results were used to suggest a mechanism for this method. Also physical, chemical and electrical characteristics of the transformer oil were measured after dechlorination and compared with the standard.

## 2. Material and methods

### 2.1. Materials

Mineral transformer oil containing 283.7 ppm mixture of Aroclor 1242, 1254 and 1260 was taken from one of the electrical substations of Isfahan, Iran. Glycerol (purity 85%), hexane (analytical grade), methylene chloride, silver nitrate and potassium chromate were obtained from Sigma-Aldrich. Sodium hydroxide, sodium carbonate, quicklime (90% pure) and zero-valent iron powder (particle size distribution: 45–63 µm) were obtained from Pars Shimi chemical company. The oxide layer on the surface of the iron powder was removed using a 3 M solution of hydrochloric acid. Aroclor mix 1 (200 µg ml^−1^ each of Aroclors 1016, 1232, 1248 and 1260 in methanol standard solution) and decachlorobiphenyl (analytical standard grade) were acquired from Sigma-Aldrich and used as calibration and surrogate standards, respectively.

### 2.2. Base-catalysed decomposition reaction process

**Figure 1.**
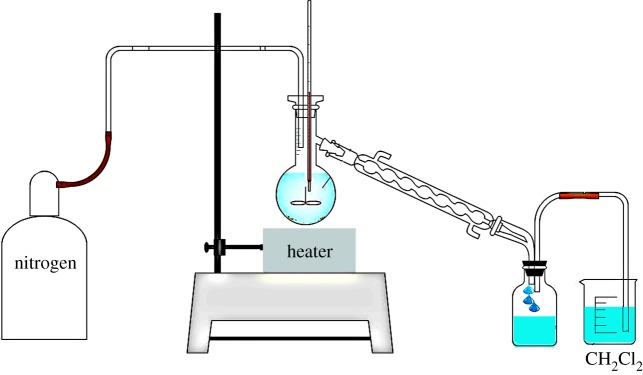
Schematic diagram of the experimental set-up made up of a reaction flask, heater, mixer, nitrogen injection system, condenser, container for holding the condensate and a cleaning vapour system.

The experimental set-up (figure 1) consisted of a 200 ml flask, a heater, a mixer, a nitrogen injection system, a condenser, a container for collecting the condensate and a container for absorption of the vapour. For all experiments, 50 g of transformer oil and 50 g of glycerol were added to the flask; and then based on each experiment, 1 mg of iron particles and/or 1 g of the base were mixed and heated at 100, 150, 200 and 250°C using the mixer speed of 300 rpm for 5 h. In addition, one blank test was performed using only the transformer oil and glycerol without any base or iron. During the first 10 min, the reaction took place in a nitrogen atmosphere to remove the oxygen from the reaction container. Some of the generated gases were condensed and released into the air after being passed through methylene chloride. At the completion of the reaction, when the flask contents reached the ambient temperature, the remaining transformer oil and condensate were weighed. The condenser, the reaction container and the remaining materials were washed with 300 ml of deionized water. Also the wash water was collected in a container and its weight and volume were measured. A quantity of 5 g of the remaining material was separated for the gas chromatography test, and the rest was washed with 200 ml of deionized water, dewatered using a centrifuge and filtered, and underwent quality tests related to transformer oils.

### 2.3. Analysis

#### 2.3.1. Standard method of silver nitrate

The quantities of chlorine ions were assessed using EPA 9253 standard. After adding 2–3 drops of potassium chromate to 25 ml of the sample and mixing it, the resulting solution was titrated using 0.1 N silver nitrate. The solution was white at first due to the precipitation of silver chloride. When the chlorine was used up, the yellow-coloured silver chromate precipitated. The endpoint of the reaction was determined by a colour change from yellow to red. The PCB dechlorination efficiency was obtained according to the following equation:

2.1 $$\text{PDE}=\left(\frac{M_{\mathrm{Cl},r}+M_{\mathrm{Cl},c}+M_{\mathrm{Cl},w}}{M_{\mathrm{Cl}}}\right)\times 100,$$

where PDE is the PCB dechlorination efficiency, *M*_Cl,r_, *M*_Cl,c_ and *M*_Cl,w_ are masses of chlorine ions in the remaining material, condensate and wash water, respectively, and *M*_Cl_ is the initial mass of chlorine ions in the transformer oil. Each test was repeated three times, and the average results are reported.

#### 2.3.2. Gas chromatography

The remaining PCBs in the transformer oil were assessed using the ASTM D-6160 standard method. For this test, a model DANI GC1000 gas chromatograph (made in Italy) with an electron capture detector (ECD) and capillary column (CP SIL-85) was used. Specifications and conditions of GC/ECD are given in table 1. Moreover, in order to prepare a 2 µg ml^−1^ surrogate standard, 1 ml of decachlorobiphenyl was poured in a 100 ml container and methanol was added to raise the volume to 100 ml. In the same way, the calibration standard was prepared.

**Table 1.** Gas chromatography specifications and conditions.

column length	50 m
column diameter	0.25 mm
column thickness	0.25 µm
injection port temperature	300°C
detector temperature	250°C
detector type	ECD
temperature programme	130°C for initial 2 min
	temperature ramp 7.5°C min^−1^ to 190°C
	temperature ramp 2.5°C min^−1^ to reach 275°C
	held for 5 min at 275°C
	cooled to 50°C

The PCB removal efficiency was obtained according to the following equation:

2.2 $$\text{PRE}=\left(1-\frac{M_{r}+M_{c}+M_{w}}{M_{0}}\right)\times 100,$$

where PRE is the PCB removal efficiency, *M*_r_, *M*_C_ and *M*_w_ are masses of PCBs in the remaining material, condensate and wash water, respectively, and *M*_o_ is the initial mass of PCBs in the transformer oil. Each test was repeated three times.

#### 2.3.3. Quality test for transformer oil

Some physical, chemical and electrical properties of the transformer oil samples including the viscosity, moisture content and dielectric loss factor were characterized by the IEC 60296 standard method before and after treatment. Viscosities were measured by a Brookfield model DV2TLV viscometer. Moisture content and dielectric loss factors were measured using the Karl Fisher titration method and a TETTEX instrument, respectively.

## 3. Results and discussion

**Figure 2.**
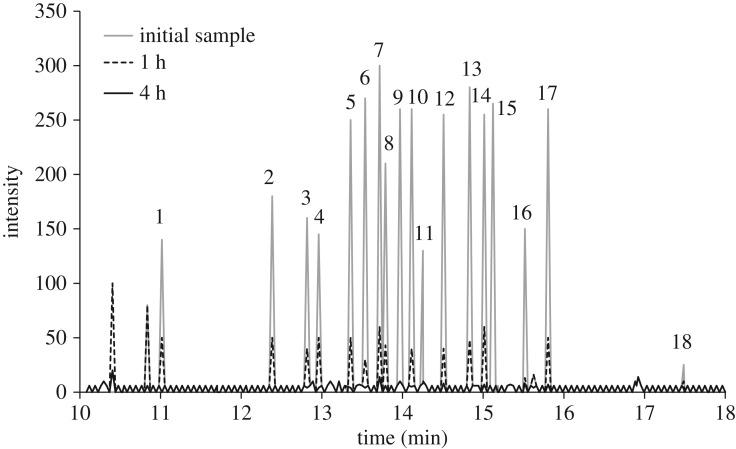
Chromatogram of detected PCBs in untreated used transformer oil, and treated oil after 1 and 4 h using iron as the catalyst. Peak index: 1—PCB 52, 2—PCB 101, 3—PCB 81, 4—PCB 77, 5—PCB 123, 6—PCB 118, 7—PCB 114, 8—PCB 153, 9—PCB 105, 10—PCB 138, 11—PCB 126, 12—PCB 167, 13—PCB 156, 14—PCB 157, 15—PCB 180, 16—PCB 169, 17—PCB 189, 18—PCB 209.

**Figure 3.**
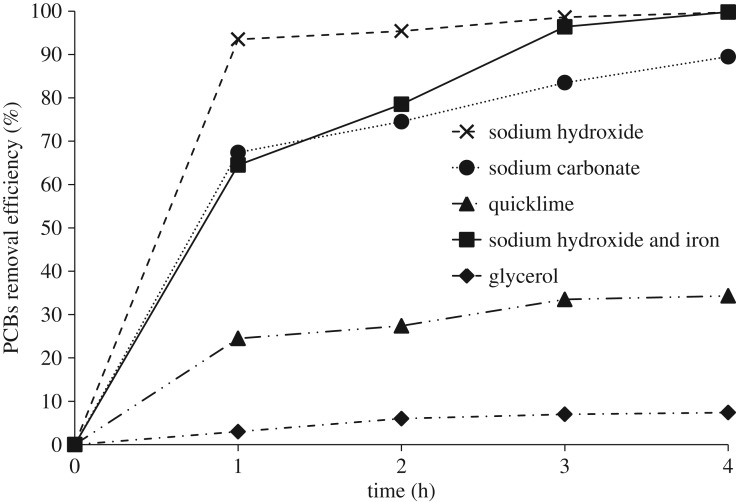
PCB removal efficiency over time for the four cases, using sodium hydroxide, sodium hydroxide and iron powder, sodium carbonate and quicklime.

**Figure 4.**
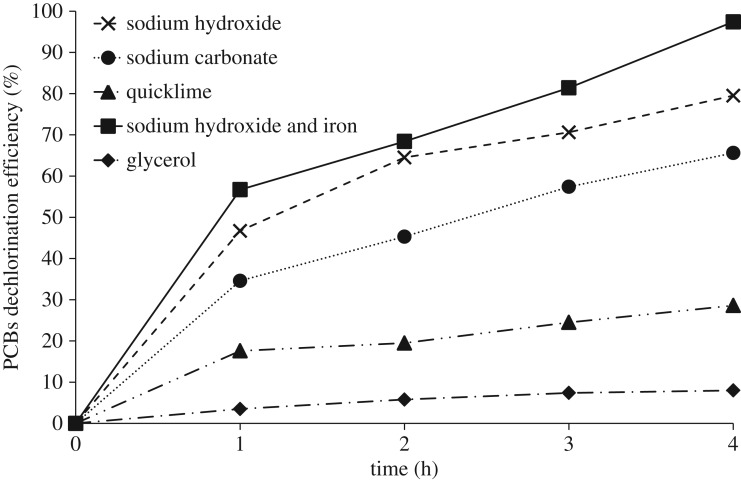
PCB dechlorination efficiency over time for the four cases, using sodium hydroxide, sodium hydroxide and iron powder, sodium carbonate and quicklime.

The chromatograms of the initial untreated oil, and oil treated with a mixture of glycerol, sodium hydroxide and iron after 1 and 4 h are compared in figure 2. No new peaks other than a peak at the very start of the chromatogram around 10.8 min, which belonged to PCB 36, was observed. The effects of time, type of base and iron on the PRE and the PDE at 250°C are presented in figures 3 and 4. As shown in figure 3, PRE values for 4 h experimental runs, using sodium hydroxide, sodium carbonate and quicklime were 99.85, 89.5 and 34.4%, respectively. Results showed that stronger bases, which had greater nucleophilic properties, were more effective in this process. These results contrasted with the finding of Hai *et al.* [17]. They expressed the base solubility in the transformer oil as the main factor. In the BCD process, PCBs are activated by the bases and intermediate materials are produced. The extent of this process depends on the strength of the base and on the temperature [16]. Besides the strength of the base, its degree of solubility in the reaction environment is one of the effective factors that determine the reaction rate. Lime had a low degree of solubility and, therefore, the minimum conversion rate was obtained when lime was used. Lime carries out dechlorination mostly through the processes of solidification and coverage of the contaminant [27].

Using sodium hydroxide, the PRE and the PDE after 1 h at 250°C reached 93.5 and 46.7%, respectively. However, these values changed to 64.5 and 56.7% when iron was used as an additional catalyst besides sodium hydroxide. Moreover, the final PRE and PDE using iron reached 99.8 and 97.4%, respectively. The reason for these different efficiency values can be explained by the different BCD mechanisms employed. When iron was not used, at least one of the chlorine atoms of the PCBs was replaced by nucleophilic groups during the first hour and produced a new compound that could not be identified as PCB by the chromatograph, although this new compound probably had chlorine atoms in its structure. After 1 h, the PRE did not change considerably. This did not mean that the dechlorination process was completed. At this time, the chlorine atoms that were not available due to the steric hindrance of other chlorine atoms and nucleophilic groups were replaced with other nucleophilic groups. The removal of chlorine from the structure of PCBs is clear from the increasing trend of PDE values in figure 4.

It was expected that the reaction would also take place through the mechanism of hydrodechlorination in the presence of the iron particles. Ye *et al*. [16] have postulated that this mechanism may proceed through electron transfer at the surface of iron particles. This is completed by oxidation of iron and breaking of hydrogen atoms from alcohol, which may replace the chlorine atoms on the PCBs. Considering the hydrodechlorination mechanism, PCBs with a high number of chlorine atoms were converted to those with low chlorine atoms when iron was used. This is reflected by the removal efficiency diagram of the case with sodium hydroxide and iron lying below that of the case with sodium hydroxide alone. However, the dechlorination curve and the final efficiency of the method in which iron was used were higher when compared with those of the method in which iron was not employed. This has been attributed to the hydrodechlorination mechanism and to the reduction in the activation energy [20,28]. The production of PCB 36 when using iron as shown in the figure 2 was indicative of the hydrodechlorination mechanism as PCB 36 was not present in the initial composition of the transformer oil. It was created by replacing the chlorine in the PCBs with a structure with a high number of chlorine atoms with hydrogen and turning them into PCBs with a low number of chlorine atoms. The hydrodechlorination mechanism when using iron was reported by Ye *et al*. [16] and Xiao *et al*. [20]. They proposed the hydrodechlorination mechanism in hexachlorobenzene degradation using the BCD method.

Using only glycerol at 250°C without base and iron, after 4 h the PRE and the PDE reached 8 and 7.4%, respectively. Without base and iron, a nucleophilic substitution mechanism was not observed [20]. Owing to the relatively low temperature, lack of iron, high boiling point of glycerol and its low nucleophilic ability, the hydrodechlorination mechanism also did not take place, and the low conversion was due to the thermal degradation of the PCBs with a low boiling point. These results were consistent with the research of Ye *et al*. [16] and contrary to the results reported by Asilian *et al*. [24]. Asilian *et al*. [24] were able to dechlorinate 26.75% of the Aroclor 1262 using PEG, although they used microwave radiation.

**Figure 5.**
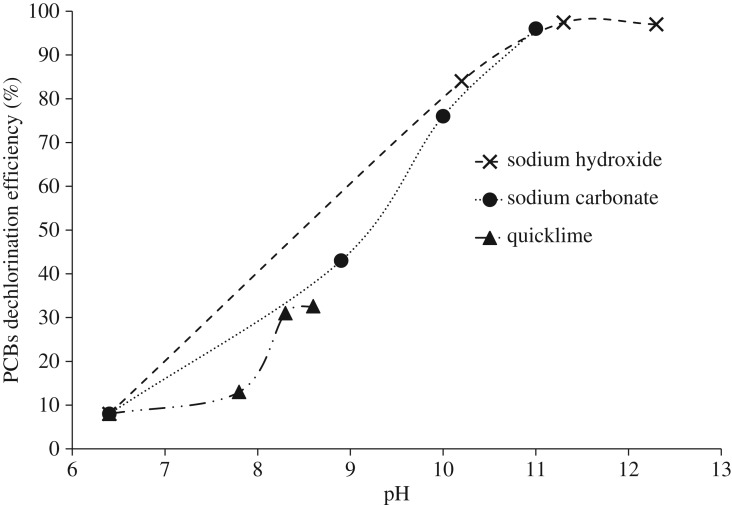
Effect of the initial pH on the PDE at 250°C for 4 h with using iron.

The effect of the initial pH on the PDE at 250°C for 4 h experimental runs with using iron is shown in figure 5. For this purpose, 0, 0.1, 1 and 10 g of each of the bases were used, and initial pH and the PDE after 4 h were measured. By using 1 g of sodium hydroxide, the initial pH and the PDE reached 11.3 and 97.4%, respectively. To achieve the same PDE with sodium carbonate, 10 g of it was required, resulting in a pH of 11. Results showed that in pH above 10, the PDE was pH-dependent and did not depend on the base type. To produce protons and anionic radicals in nucleophilic reactions, strong bases are needed, and the higher the pH, the greater is the ability to remove protons from the environment [10,13]. Therefore, at high pH, the proton is easily removed from glycerol and, given the uniformity of the cation in the structure of sodium hydroxide and sodium carbonate, if the temperature is sufficient, the reaction is carried out with high efficiency. Owing to the low solubility and reduced solubility with increasing temperature, quicklime did not have the necessary efficiency in this method [14]. Using iron without bases, for 4 h experimental runs at 250°C, the PDE was 8%. This low conversion is due to the low temperature because iron without bases requires high temperature for hydrodechlorination reactions to proceed [13].

**Figure 6.**
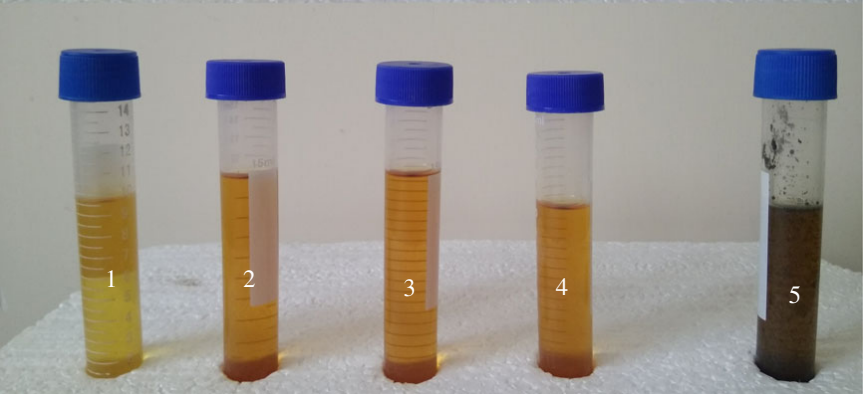
Colour of the transformer oil after treatment at different temperatures: (1) initial sample, (2) 100°C, (3) 150°C, (4) 200°C and (5) 250°C.

With increasing the temperature from 150 to 250°C, the PDE increased from 54.4% to 97.4% when sodium hydroxide and iron powder were used (table 2). At temperatures below 100°C, the conversion rate was low because of the high viscosity of glycerol, which reduced the diffusion rate of the bases towards transformer oil molecules. Although the conversion rate reached its maximum at 250°C, physical and electrical properties of the transformer oil at this temperature changed in a way that rendered it out of specified standards. Figure 6 shows the effect of temperature and reaction products of PCB dechlorination on the colour of transformer oil at temperatures in the range of 100–250°C. With a temperature up to 200°C, no noticeable colour change in the oil was observed. This was an indicator of the physical health of the oil. By increasing the temperature up to 250°C, oxidation of oil occurred and degradation in the structure of the oil caused a change in its colour. The oxidation particles had a very destructive effect on the insulation loss factor of the oil [29].

**Table 2.** Effect of temperature on the PDE and properties of transformer oil.

temperature	PDE	viscosity at 100°C (mm^2^ s^−1^)	moisture content (mg dm^−3^)	dielectric loss factor at 90°C
IEC 60296 standard limits		10.23	30	0.005
initial sample		2.54	13.0	0.0014
50	13.4	2.54	13.0	0.0017
100	38.0	2.56	12.7	0.0017
150	54.4	2.55	13.0	0.0021
200	89.4	2.55	13.2	0.0018
250	97.4	2.70	16.0	0.0064

As shown in table 2, the insulation loss factor of the treated oil at 250°C was not compliant with applicable standards. As the solid particles and water were removed from the treated oil by filtration, these changes cannot be attributed to these factors, and may only be thought to be caused by destruction of the structure of the mineral oil at high temperatures. By applicable standards, oils with these specifications are not suitable for use in transformers and more clearing operations are required. At 200°C, although the conversion rate was lower, physical and electrical standards were satisfactory, and the levels of PCBs remaining in the oil with this conversion rate were acceptable by many standards.

**Figure 7.**
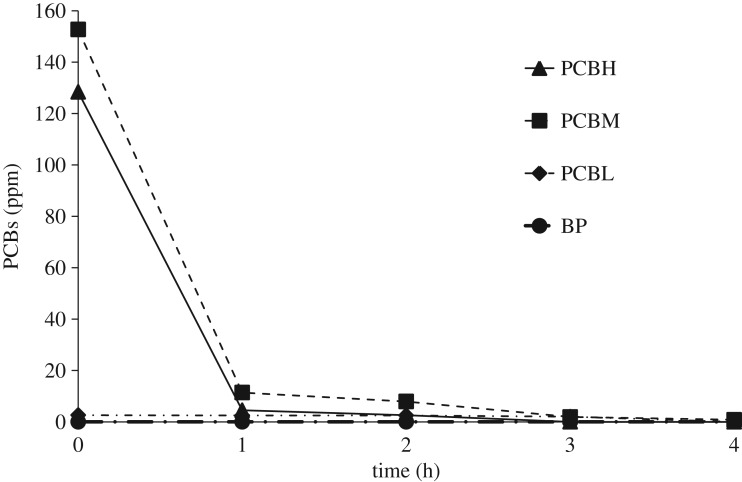
Different kinds of PCB congener mixtures at 250°C with respect to time, when iron was not used.

**Figure 8.**
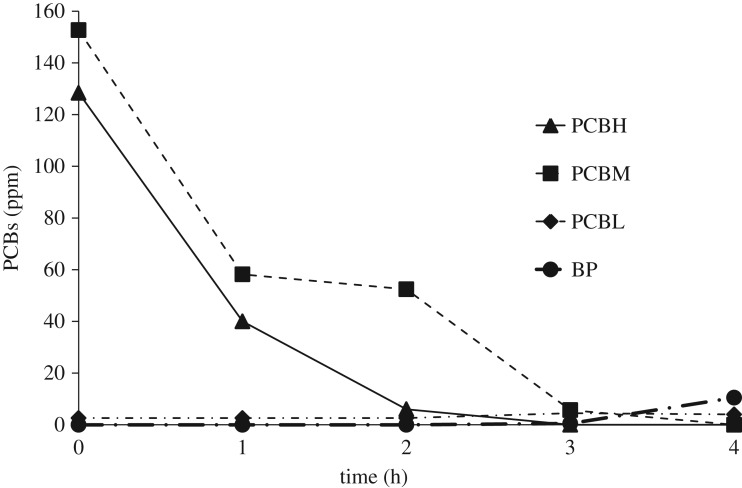
Different kinds of PCB congener mixtures at 250°C with respect to time, when iron was used.

Changes in concentrations of PCBH (PCBs with seven or more chlorine atoms), PCBM (four to six chlorine atoms), PCBL (less than three chlorine atoms) and BP (biphenyl) at 250°C with respect to time are shown in figures 7 and 8. When iron was not used (figure 7), concentrations of all three types of PCBs declined with time, reaching almost a zero value after an hour. As previously explained, the nucleophilic group replaced chlorine atoms with less steric hindrance by attacking them. At that moment, though a substantial number of chlorine ions remained in the structure of the PCBs (which were separated later on), this compound could no longer be identified using gas chromatography. However, when iron was used (figure 8), part of the dechlorination process proceeded through the hydrodechlorination mechanism, and PCBs with a larger number of chlorine atoms were converted into those with fewer ones. As shown in figure 8, PCBs with a small number of chlorine atoms increased to some peak values before decreasing again. On the other hand, concentrations of the PCBs with a larger or average number of chlorine atoms were decreasing all the time. In this method, production of biphenyl confirmed the presence of the hydrodechlorination mechanism.

Mass balance of chlorine atoms for experiments using sodium hydroxide (without iron) is shown in table 3. After 1 h of reaction, while 93.5% of the PCBs was removed, the total recovery, as calculated by the equation in the footnote of table 3, was 53%. This confirmed the nucleophilic substitution mechanism for experimental runs using sodium hydroxide without iron. It shows that nucleophilic groups had replaced some chlorine atoms in the PCB structure and prevented their identification. For the same experiments, after 4 h of reaction, the total recovery was 80%, with 99.3% of PCB removal. The results showed that despite the fact that all of the PCBs dechlorinated, there was still 55 µmol of chlorine in the by-products, which was not separated.

**Table 3.** Mass balance of chlorine atoms without using iron.

	PCBs in transformer oil		chloride ion		
time (h)	amounts (µmol)^a^	percent (%)	amounts (µmol)^b^	percent (%)	recovery (%)^c^
0	275 ± 5				
1	18 ± 3	6.54	128 ± 1	46.5	53
2	15 ± 1	5.45	177 ± 5	64.3	69.45
3	4 ± 2	1.45	194 ± 4	70	71.45
4	2 ± 2	0.7	218.6 ± 3	79.3	80

^a^The amounts are the total chlorine atoms of PCBs in the reaction system.

^b^The amounts are the total chlorine ions in the reaction system.

^c^The total recovery ratio = (*a* + *b*)/275.

In this method, dechlorination was done according to equations (3.1) to (3.4). Equations (3.3) and (3.4) describe hydrodechlorination and nucleophilic substitution mechanisms, respectively.

3.1 $$\begin{array}{rl} \text{NaOH} & \to \text{N}{\text{a}}^{+}+\text{O}{\text{H}}^{-}, \end{array}$$

3.2 $$\begin{array}{rl} \text{MOH} & \to \text{M}{\text{O}}^{-}+{\text{H}}^{+}, \end{array}$$

3.3 $$\begin{array}{rl} \text{MOH}+\text{O}{\text{H}}^{-} & \to \text{MO}{\text{O}}^{-}+{\text{H}}_{2} \end{array}$$

3.4 $$\begin{array}{rl} \;\text{and}\;\text{MOH}+\text{NaOH} & \to \text{MONa}+{\text{H}}_{2}\text{O}. \end{array}$$

**Figure 9.**
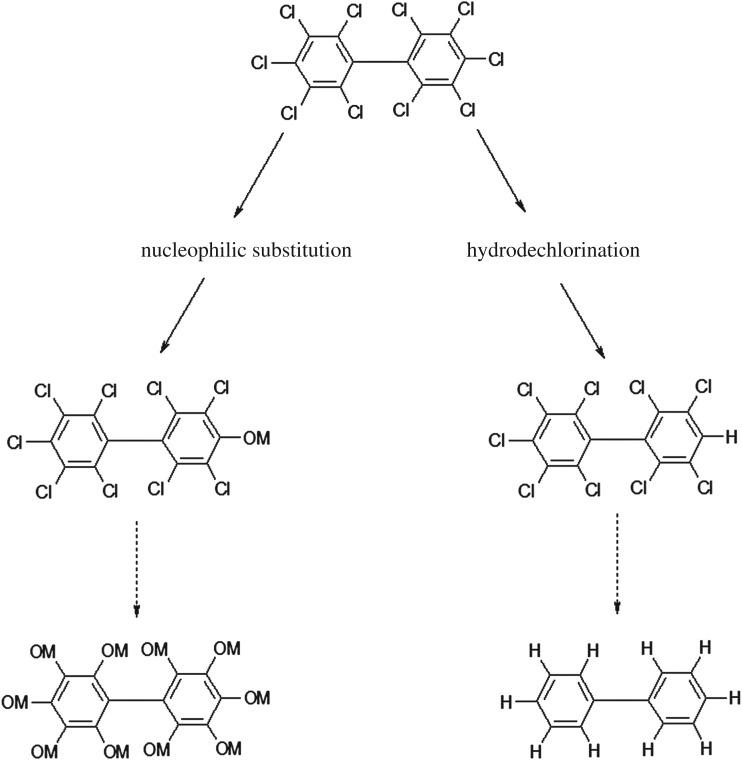
Dechlorination pathways.

In this BCD process, hydrogen ions which were removed from alcohol, and hydroxide ions which were removed from base, produced water, alkoxide and hydrogen. Then dechlorination was carried out through the two routes of using hydrogen or alkoxide. The final product in the hydrodechlorination mechanism, which has been investigated in many studies, was biphenyl [13,30]. Figure 9 shows the nucleophilic substitution and hydrodechlorination mechanisms. In the BCD method in which glycerine without iron powder is used, the main reaction takes place through nucleophilic substitution. Hai *et al*. [17] and Xiao *et al*. [20] proposed a combined mechanism in their research while Kamarehie *et al*. [10] only considered nucleophilic substitution.

## 4. Conclusion

In the present research, dechlorination of PCBs was studied employing the BCD method in which glycerol, sodium hydroxide and iron particles were used. Using this method at 250°C for 4 h, a PCB removal efficiency of 99.8% and dechlorination efficiency of 97.4% were achieved. However, the treated oil lacked the necessary standards to be reused in the transformer. On the other hand, carrying out the reactions at 200°C led to the treated oils conforming to standard physical and chemical properties, and achieving the acceptable PDE of 89.4%. In this method, it was found that the dechlorination efficiency is dependent on the pH, and was not dependent on the base type. Furthermore, without using bases, iron alone was not effective in PCB dechlorination. The mechanism related to this method is a multistage mechanism. Without the presence of iron, it proceeds through nucleophilic substitution. However, a combined mechanism of hydrodechlorination and nucleophilic substitution is active in the presence of iron as the catalyst. In this method, results of the simple chromatography test alone are not interpretable and may be misleading. Besides chromatography, the amount of free chlorine ions must be measured. Although this is a very cost-effective method, more research is yet required for optimizing the temperature and catalysts. Moreover, many points related to stability and toxicity of the obtained product, and the precise mechanism of the reaction, remain to be studied.

## Supplementary Material

Data file for Figures 3,4,5,7,8

## Supplementary Material

Data file for Figure-2

## Data accessibility

The datasets supporting this article have been uploaded as part of the electronic supplementary material.
